# A Meta-Analysis of Survival Outcomes Following Reoperation in Recurrent Glioblastoma: Time to Consider the Timing of Reoperation

**DOI:** 10.3389/fneur.2019.00286

**Published:** 2019-03-26

**Authors:** Yu-Hang Zhao, Ze-Fen Wang, Zhi-Yong Pan, Dominik Péus, Juan Delgado-Fernandez, Johan Pallud, Zhi-Qiang Li

**Affiliations:** ^1^Department of Neurosurgery, Zhongnan Hospital, Wuhan University, Wuhan, China; ^2^Department of Physiology, School of Basic Medical Sciences, Wuhan University, Wuhan, China; ^3^Department of Neurosurgery, University Hospital Zurich, Zurich, Switzerland; ^4^Division of Neurosurgery, University Hospital La Princesa, Madrid, Spain; ^5^Department of Neurosurgery, Sainte-Anne Hospital, Paris, France; ^6^Paris Descartes University, Sorbonne Paris Cité, Paris, France

**Keywords:** glioblastoma, recurrence, reoperation, time-dependent covariate, survival

## Abstract

**Background:** Glioblastoma multiforme (GBM) inevitably recurs, but no standard regimen has been established for recurrent patients. Reoperation at recurrence alleviates mass effects, and the survival benefit has been reported in many studies. However, in most studies, the effect of reoperation timing on survival benefit was ignored. The aim of this meta-analysis was to investigate whether reoperation provided similar survival benefits in recurrent GBM patients when it was analyzed as a fixed or time-dependent covariate.

**Methods:** A systematic literature search of PubMed, EMBASE, and Cochrane databases was performed to identify original articles that evaluated the associations between reoperation and prognosis in recurrent GBM patients.

**Results:** Twenty-one articles involving 8,630 patients were included. When reoperation was considered as a fixed covariate, it was associated with better overall survival (OS) and post-progression survival (PPS) (OS: HR = 0.66, 95% CI 0.61-0.71, *p* < 0.001, *I*^2^ = 0%; PPS: HR = 0.70, 95% CI 0.57–0.88, *p* < 0.01, *I*^2^ = 70.2%). However, such a survival benefit was not observed when reoperation was considered as a time-dependent covariate (OS: HR = 2.19, 95% CI 1.47–3.27, *p* < 0.001; PPS: HR = 0.95, 95% CI 0.82–1.10, *p* = 0.51, *I*^2^ = 0%). The estimate bias caused by ignoring the time-dependent nature of reoperation was further demonstrated by the re-analysis of survival data in three included studies.

**Conclusions:** The timing of reoperation may have an impact on the survival outcome in recurrent GBM patients, and survival benefits of reoperation in recurrent GBM may be overestimated when analyzed as fixed covariates. Proper analysis methodology should be used in future work to confirm the clinical benefits of reoperation.

## Introduction

Glioblastoma multiforme (GBM) is the most common malignant brain tumor in adults, and the tumor recurs in nearly all patients, with a median survival of 12–15 months from initial diagnosis (1). Although a standard STUPP protocol has been widely used for newly diagnosed GBM (2, 3), no such regimen is fully established for recurrent patients (4–6). Reoperation is an increasingly common treatment option for recurrent GBM patients due to useful symptom relief. In the past decade, some novel intraoperative techniques, such as intraoperative imaging, fluorescence-guided surgery and intraoperative stimulation mapping, have been developed to maximize tumor cytoreduction and minimize surgical morbidity, which significantly improved the surgical management of glioma patients (7).

In addition to improvements in neurologic symptoms and quality of life, survival time is an important indicator in assessing the prognostic value of treatment in tumor diseases. However, the survival benefit of reoperation in recurrent GBM patients remains controversial, owing to a lack of high-level evidence from high-quality randomized trials, prospective studies, and meta-analysis (8, 9). The beneficial effect of repeat resection on survival has been reported in recurrent GBM patients in some studies (10–18), but a reverse effect of reoperation was also shown in other studies (19–27). Of these studies, the survival benefit of reoperation was evaluated by overall survival (OS) or post-progression survival (PPS). Survival outcome in GBM patients is affected by many confounding factors, including age, Karnofsky performance status (KPS), tumor volume, tumor location, treatment schedule, resection extent, and O^6^-methylguanine-DNA methyltransferase (*MGMT*) promoter methylation as well as *IDH1* mutation status. These confounding factors are incorporated into survival analyses as fixed covariates or time-dependent covariates. Fixed covariates such as sex remain unchanged during the study. Time-dependent covariates, also called time-varying covariates, may be unknown at the start of observation and may change their value over time. Reoperation is a time-dependent factor in nature but is defined as a fixed covariate in most of the GBM reoperation literature (10–19, 23, 24, 26, 28–30). Improper analysis of a time-dependent covariate as a fixed value always leads to estimate bias, favoring longer survival (21, 31, 32). Recently, a published meta-analysis demonstrated the survival benefit of reoperation in recurrent GBM patients; however, the time-dependent characteristic of reoperation was neglected (33).The aim of this study was to systematically review the survival benefit of reoperation in recurrent GBM patients when analyzed by time-dependent and non-time-dependent methods.

## Methods

### Search Strategy

A comprehensive literature review was conducted to identify the articles covering the association of reoperation with prognosis in GBM from PubMed, EMBASE and the Cochrane Library. The present review was conducted according to the criteria outlined in the PRISMA (Preferred Reporting Items for Systematic reviews and Meta-Analyses) guideline (34). The articles enrolled in this analysis were published between January 1, 2005 and September 1, 2018. The Mesh terms used for literature search included “Glioblastoma,” “Recurrenc,” and “Reoperation.” The following non-Mesh terms were also used: (1) “Glioblastomas,” “Astrocytoma, Grade IV,” “Astrocytomas, Grade IV,” “Grade IV Astrocytoma,” “Grade IV Astrocytomas,” “Glioblastoma Multiforme,” “Giant Cell Glioblastoma,” “Giant Cell Glioblastomas,” “Glioblastoma, Giant Cell,” “Glioblastomas, Giant Cell”; (2) “Recurrences,” “Recrudescence,” “Recrudescences,” “Relapse,” “Relapses”; (3) “Surgical Revision,” “Surgery, Repeat,” “Revision, Surgical,” “Revision Surgery,” “Revision Surgeries,” “Surgery, Revision,” “Repeat Surgery,” “Revision, Joint,” “Joint Revision,” “Repeat operation,” “Repeat resection,” and “Second surgery,” Reviews as well as the references of the included studies were also checked to avoid the omission of relevant publications. The eligible studies were restricted to human beings.

### Inclusion and Exclusion Criteria

Articles that met the following criteria were included in this study: (1) studies investigating the relationship between reoperation and survival in recurrent GBM patients; (2) multivariate Cox-proportional hazard regression models were used to minimize the bias from baseline confounding factors; and (3) hazard ratios (HR) and 95% confidence intervals (CI) for survival time were available directly. We excluded publications as follows: (1) reviews, laboratory research, and animal experiments; and (2) studies published only in abstract.

### Study Selection and Data Extraction

Study selection was independently performed by two authors, and disagreements were resolved through discussion. The following data were extracted: the first author's name, country of origin, publication year, number of patients, adjustment method, and outcomes (including HRs and 95% CIs).

### Quality Assessment

The Newcastle Ottawa Scale (NOS) was used to assess the quality and risk of bias of the included studies (35). Three main aspects of quality were evaluated, including study selection, comparability, and study outcome. The assessment of the included studies was independently conducted by two authors. High scores indicate high quality and low risk of bias.

### Data Analysis

The statistical analysis was performed with STATA 12.0 software (StataCorp, College Station, TX, USA). The statistical heterogeneity among studies was assessed by a Q-test and *I*^2^ statistics. If there was significant heterogeneity, the random effect model was used to estimate the pooled HR; otherwise, the fixed effect model was used. Publication bias was assessed by funnel plots and Egger's test. Sensitivity analysis by deleting each included study was conducted to assess the overall robustness of the meta-analysis results.

To illustrate the estimate bias that occurs when a time-dependent covariate is treated as known at the start of the study, the survival curves of three included studies (12, 15, 25), in which reoperation was considered as a fixed covariate in the multivariate Cox model, were plotted and reanalyzed by three methods. The traditional Kaplan-Meier method treats the covariate as a fixed value at the start of the study. The landmark method selects a fixed time point after the start of the study as a landmark for conducting the analysis, and it is a valid method to test whether the survival is related to patients' status at a specific time point (36, 37). We selected the 50th percentile and 75th percentile of time between the first and second surgery as the landmark time, as described by Goldman (21). Only patients alive at the landmark time were included in the analysis, and they were separated into reoperation and non-reoperation groups according to their resection status by the landmark time. Then, group comparisons were conducted with the Breslow test. Survival data were also analyzed by the Simon-Makuch method (38), which takes into account the change in covariate status over time and properly represents the effect of a time-dependent covariate on survival. In the Simon-Makuch method, patients move from non-reoperation group to reoperation group at the time their reoperation occurs. Therefore, the number of patients changes over time.

## Results

### Characteristics of the Studies

**Figure 1:**
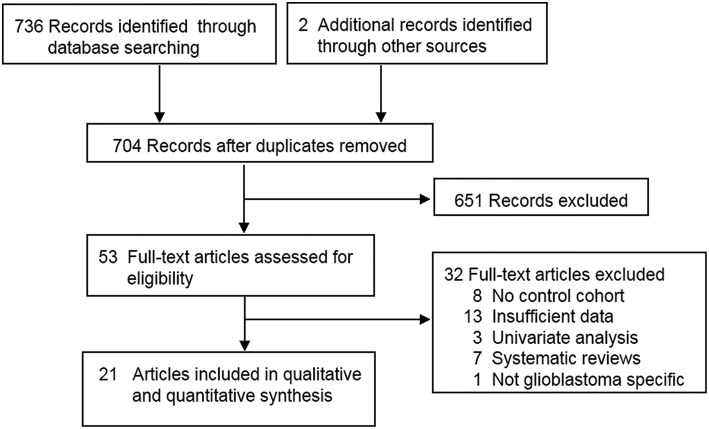
PRISMA flow diagram of study selection.

The flow chart of the literature selection is presented in Figure 1. A total of 738 articles were screened, and 21 articles comprising 8,630 patients were identified (10–28, 39, 40). Of these publications, two articles comprising two independent trials (marked with I and II in the figures and tables) were extracted as four studies (20, 22). Therefore, a total of 23 studies were included in this analysis. The characteristics of all included studies are summarized in Tables 1, 2. The quality assessment showed that all studies were of high quality (see Supplemental Table S1).

**Table 1:** Characteristics of included studies for overall survival analysis.

**Name**	**Study type**	**Country**	**Patients number**	**Treatment**
Chaichana et al. (A) (16)	Retrospective	America	522	Reoperation+/RT+/chemo vs. /RT+/chemo
Chaichana et al. (B) (16)	Retrospective	America	395	Multiple reoperation+/RT+/chemo vs. /RT+/chemo
Chaichana et al. (C) (16)	Retrospective	America	369	Third reoperations+/RT+/chemo vs. /RT+/chemo
Chen et al. (A) (11)	Retrospective	America	3882	Reoperation+/chemo+/RT vs. / RT+/chemo
Chen et al. (B) (11)	Retrospective	America	3575	Multiple reoperation+/chemo+/RT vs. RT+/chemo
Delgado-Fernandez et al. (12)	Retrospective	Spain	121	Reoperation+ RT+ chemo vs. RT+/chemo
Goldman et al. (21)	Retrospective	America	163	Reoperation+/RT+/chemo vs. /RT+/chemo
Ortega et al. (A) (23)	Retrospective	America	177	Reoperation+ RT+/chemo vs. RT+/chemo
Ortega et al. (B) (23)	Retrospective	America	108	Multiple reoperation+ RT+/chemo vs. RT+/chemo
Skeie et al. (13)	Retrospective	Norway	51	Reoperation+ RT+/chemo vs. RT+/chemo
Stark et al. (17)	Retrospective	Germany	122	Reoperation+/chemo +/RT vs. /chemo+/RT
Tugcu et al. (26)	Retrospective	Turkey	50	Reoperation+/RT vs. /RT
Tully et al. (40)	Retrospective	America	204	Reoperation+/chemo+/ RT vs. /chemo+/RT
Woernle et al. (25)	Retrospective	Switzerland	98	Reoperation+/chemo vs. /chemo

*RT, radiotherapy; chemo, chemotherapy*.

**Table 2:** Characteristics of included studies for post-progression survival analysis.

**Name**	**Study type**	**Country**	**Patients number**	**Treatment**
Azoulay et al. (18)	Retrospective	Canada	78	Reoperation+chemo+/ RT vs. chemo +/ RT
Boiardi et al. (10)	Retrospective	Italy	211	Reoperation+chemo vs. chemo
Clarke et al. (19)	Prospective	America	593	Reoperation+/chemo vs. /chemo
Filippini et al. (I) (20)	Retrospective	Italy	452	Reoperation+/chemo +/ RT vs. /chemo+/RT
Filippini et al. (II) (20)	Retrospective	Italy	435	Reoperation+/chemo +/ RT vs. /chemo+/RT
Nava et al. (I) (22)	Prospective	Italy	203	Reoperation+/chemo +/ RT vs. /chemo+/RT
Nava et al. (II) (22)	Prospective	Italy	303	Reoperation+/chemo +/ RT vs. /chemo+/RT
Skeie et al. (13)	Retrospective	Norway	51	Reoperation+/chemo+RT vs. RT+/chemo
Suchorska et al. (24)	Prospective	Germany	105	Reoperation+chemo vs. chemo
Wann et al. (14)	Retrospective	Australia	117	Reoperation+/chemo vs. /chemo
Zanello et al. (15)	Retrospective	France	777	Reoperation+/RT+/chemo vs./chemo+/RT
Sastry et al. (27)	Retrospective	America	368	Reoperation+chemo+/RT vs. chemo +/RT
Kim et al. (39)	Retrospective	Korea	38	Reoperation+chemo+RT
McGirt et al. (28)	Retrospective	America	285	Reoperation+chemo+RT

*RT, radiotherapy; chemo, chemotherapy*.

### Publication Bias

Publication bias was evaluated by Egger's test. No publication bias was observed in the analysis of OS (*p* = 0.69) or PPS (*p* = 0.15; see Supplemental Figure S1).

### Sensitivity Analysis

Sensitivity analysis was conducted by sequentially omitting individual studies to assess whether a single study might significantly affect the overall results. Sensitivity analysis showed no apparent variations in the pooled HR of OS or PPS (Supplemental Figure S2), supporting the robustness of the primary findings.

### Impact of Reoperation at Recurrence on OS

**Figure 2:**
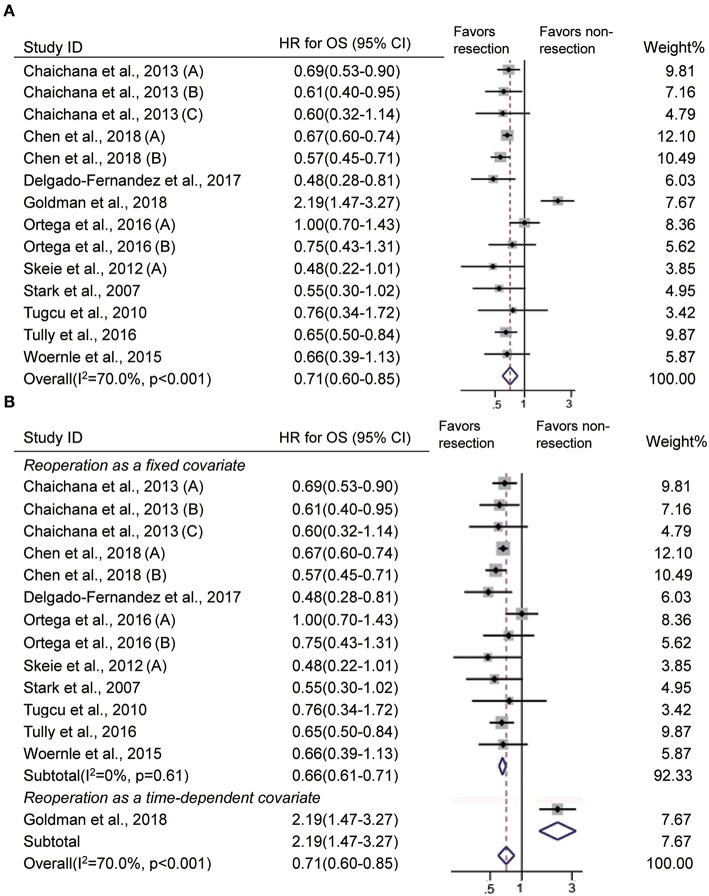
Forest plots showing pooled results of overall survival analysis in recurrent patients with vs without reoperation **(A)** and when reoperation was considered as a fixed covariate vs. as a time-dependent covariate **(B)**.

In 10 studies (11–13, 16, 17, 21, 23, 25, 26, 40), survival was defined as the time from the first surgery to death or the end of follow-up, and it indicates OS. Our analysis showed that recurrent patients who underwent reoperation had significantly prolonged OS than those who did not (HR = 0.71, 95% CI 0.60–0.85, *p* < 0.001, *I*^2^ = 70%, Figure 2A). GBM patients may undergo more than one resection at recurrence. The impact of one reoperation and multiple reoperations on OS was reported in 10 studies (11–13, 16, 17, 21, 23, 25, 26, 40) and 3 studies (11, 16, 23), respectively. Our analysis showed that both one resection and multiple resections upon recurrence significantly extended OS in patients with GBM (one reoperation: HR = 0.75, 95% CI 0.59–0.95, *p* = 0.02, *I*^2^ = 77.3%; multiple reoperation: HR = 0.59, 95% CI 0.50–0.71, *p* < 0.001, *I*^2^ = 0%, Supplemental Figure S3).

In these studies, reoperation was incorporated into the multivariate Cox model as a fixed covariate in 9 studies (11–13, 16, 17, 23, 25, 26, 40) and as a time-dependent covariate in one study (21). The difference between the covariates was whether the timing of reoperation was taken into consideration. Then, we evaluated whether reoperation had a similar impact on OS when analyzed with time-dependent and non-time-dependent methods. Reoperation was associated with longer OS when the timing was ignored (HR = 0.66, 95% CI 0.61–0.71, *p* < 0.001, *I*^2^ = 0%, Figure 2B) and was not associated with better OS when timing was taken into account (HR = 2.19, 95% CI 1.47–3.27, *p* < 0.001, Figure 2B).

**Figure 3:**
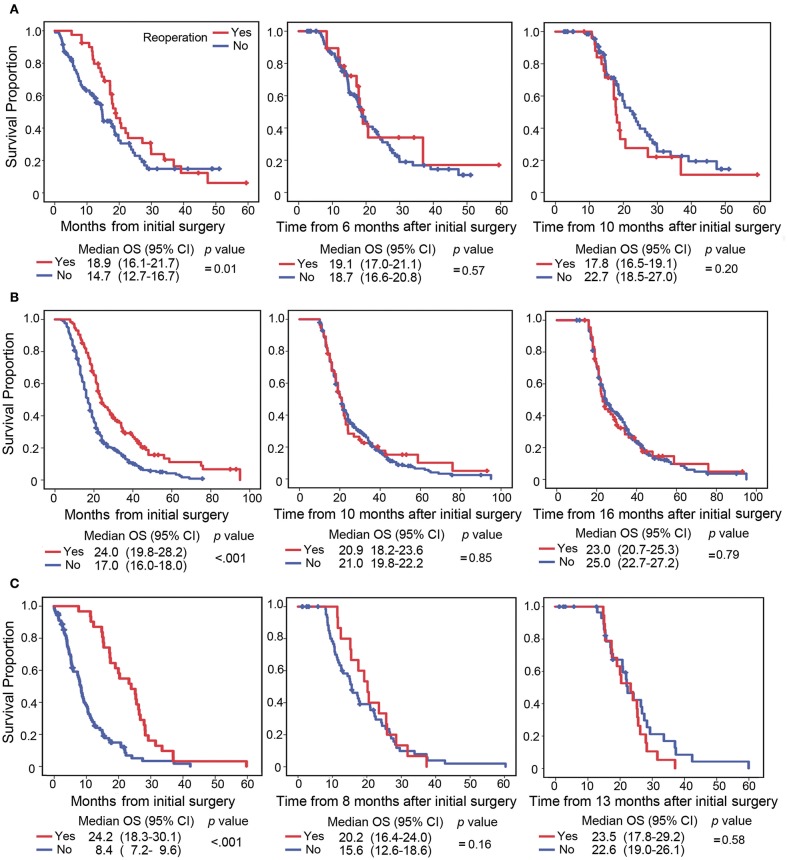
Survival curve plotted by the Kaplan-Meier and landmark methods. The survival data from the studies of Woernle et al. (25) **(A)**, Zanello et al. (15) **(B)**, and Delgado-Fernandez et al. (12) **(C)** were reanalyzed by the Kaplan-Meier (left) and landmark methods (middle and right). The Kaplan-Meier curve started at the initial diagnosis (left), and the landmark curve started at the 50th percentile (middle) and 75th percentile (right) of time between first and second surgery. In the landmark method, patients were considered to have reoperation if their reoperation occurred by the landmark time and were considered to not have reoperation if they did not undergo reoperation or their reoperation occurred after the landmark time.

Given the sparse number of studies with time-dependent analyses, more evidence was needed to demonstrate the estimate bias occurring in survival analysis, where a time-varying covariate is treated as a time-fixed one. The survival data of the three included studies (12, 15, 25) were reanalyzed by the traditional Kaplan-Meier method, landmark method and Simon-Makuch method. Traditional Kaplan-Meier curves showed that patients with reoperation had prolonged OS compared with those without reoperation (Figure 3). However, the landmark analysis showed that survival was not associated with better OS at two specific landmark times (Figure 3). Similarly, survival analysis by the Simon-Makuch method did not shown long OS in patients with reoperation (Woernle et al: HR = 1.45, 95% CI 0.91–2.28, *p* = 0.12; Zanello et al: HR = 0.99, 95% CI 0.77–1.26, *p* = 0.91; Delgado-Fernandez et al: HR = 0.79, 95% CI 0.49–1.28, *p* = 0.91). These results indicated that ignoring the time-dependent nature of reoperation might lead to estimate bias.

### Impact of Reoperation at Recurrence on PPS

**Figure 4:**
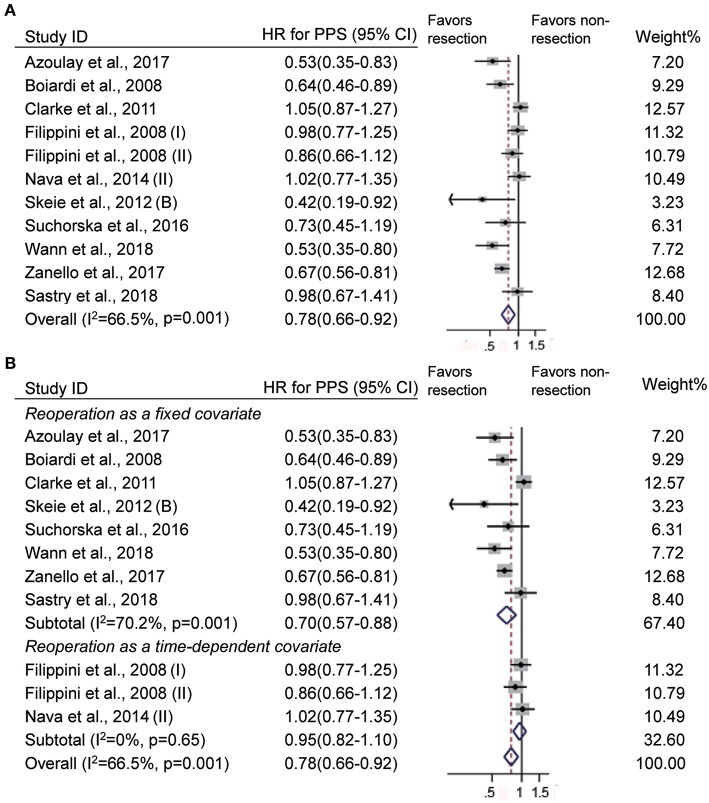
Forest plots showing pooled results of post-progression survival analysis in patients with vs. without reoperation **(A)** and when reoperation was considered as a fixed covariate vs. as a time-dependent covariate **(B)**.

When deciding the clinical benefits of treatment at tumor recurrence, to some extent, PPS may be more appropriate for evaluating the survival effect of treatment. In 14 studies (10, 13–15, 18–20, 22, 24, 27, 28, 39), survival was defined as the time from the first recurrence to death or the end of follow-up, and it indicates PPS. There were 11 studies that evaluated the impact of reoperation on PPS (10, 13–15, 18–20, 22, 24, 27). Consistent with OS, reoperation at recurrence was associated with improved PPS (HR = 0.78, 95% CI 0.66–0.92, *p* < 0.01, *I*^2^ = 66.5%, Figure 4A). In these studies, reoperation was incorporated as a fixed covariate in 8 studies (10, 13–15, 18, 19, 24, 27) and as a time-dependent covariate in 3 studies (20, 22). When the timing of reoperation was ignored, reoperation was also associated with long PPS (HR = 0.70, 95% CI 0.57–0.88, *p* < 0.01, *I*^2^ = 70.2%, Figure 4B). However, patients with reoperation had similar PPS as those without reoperation when the timing of reoperation was taken into account (HR = 0.95, 95% CI 0.82–1.10, *p* = 0.51, *I*^2^ = 0%, Figure 4B).

**Figure 5:**
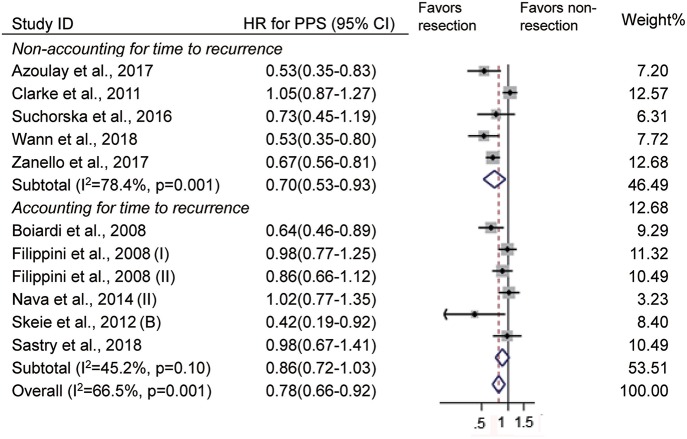
Forest plots for post-progression survival analysis when accounting for vs. not accounting for the time to first recurrence.

Of the 11 studies, 6 studies incorporated the time from the initial diagnosis to first the recurrence into the multivariate Cox model (10, 13, 20, 22, 27). When the time to first recurrence was not considered, patients who underwent reoperation had a prolonged PPS compared with those who did not (HR = 0.70, 95% CI 0.53–0.93, *p* = 0.02, *I*^2^ = 78.4%, Figure 5). However, comparable PPS was observed between patients with and without reoperation when the time to first recurrence was taken into account (HR = 0.86, 95% CI 0.72–1.03, *p* = 0.10, *I*^2^ = 45.2%, Figure 5).

**Figure 6:**
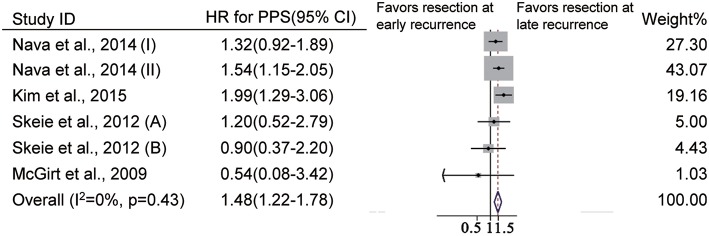
Forest plots for post-progression survival analysis in patients undergoing reoperation after earlier recurrence vs. later recurrence.

We further evaluated the impact of time to recurrence on PPS in GBM patients who underwent reoperation. Of the included studies, the cutoff value of the earlier recurrence was defined as 9 months in three studies (22, 39), as 10 months in one study (13) and as 12 months in one study (28). Among GBM patients who underwent reoperation, patients with earlier recurrence had a higher risk of death than those with later recurrence (HR = 1.42, 95% CI 1.22–1.7, *p* < 0.001, *I*^2^ = 0%, Figure 6).

## Discussion

Neurosurgeons always pay close attention to OS and progression-free survival (PFS) when evaluating the clinical benefits of the primary regimen in tumor patients. Due to the inherent bias arising from prolonged pre-progression survival in patients who undergo repeat resections, the survival benefit of reoperation at tumor progression may be overestimated by measuring OS (41). PPS may be more appropriate to evaluate the survival effect of repeat resections at tumor recurrence. In this study, we conducted a comprehensive analysis to evaluate the association of reoperation with OS and PPS in recurrent GBM patients.

We first analyzed all the included studies and found that reoperation improved both OS and PPS in recurrent patients. In addition, both one resection and multiple resections upon recurrence significantly prolonged OS. These results seem to strongly support surgical management in recurrent GBM patients. However, further analysis showed that better OS and PPS were observed only when reoperation was taken into account as a fixed covariate. We noticed that reoperation was considered a time-dependent covariate in few studies (20–22). To further demonstrate the reliability of our results, survival data in the three included studies, in which the timing of reoperation was not considered, were reanalyzed by treating reoperation as a fixed and a time-dependent covariate, respectively. Consistently, the association of reoperation with improved OS was not found when analyzed by either the landmark or Simon-Makuch method, in which the time-dependent effects of reoperation were considered. In fact, time from initial diagnosis to recurrence or reoperation is unpredictable and variable in GBM patients. Therefore, reoperation is a time-dependent variable in nature. Time-dependent methodology has been well-established in survival analysis (42) and had been used in prognosis analysis of cancers, such as colon cancer (43), breast cancer (44) and leukemic evolution (45). Previous studies reported that ignoring time-dependent bias inevitably overestimated the survival benefit and led to results supporting longer survival (21, 31, 32, 46). Therefore, the timing of reoperation should be included to evaluate the impact of reoperation on survival in GBM patients.

The importance of the timing of reoperation on survival is also supported by the high risk of death in GBM patients with earlier recurrence. Our results showed that the time to recurrence had a significant impact on the prognostic value of reoperation. The time of recurrence means, to some extent, the time of reoperation. Therefore, the high risk of death in tumor patients with earlier recurrence also underscored the importance of time-dependent methodology in survival analysis. GBM patients who experience earlier recurrence usually have more a progressive phenotype of the tumor and/or molecular risk factors such as *MGMT* promoter unmethylation. These tumor histological and molecular characteristics are independently associated with worse survival. Both tumor-related and treatment-related factors could affect clinical outcomes, but the presence of tumor-related characteristics may have a greater effect on tumor progression and survival than reoperation itself. When the time to recurrence or reoperation was not considered, the impact of some tumor-related characteristics might be ignored in survival analysis. These understandings can help us to correctly interpret the worse PPS observed in patients with early recurrence. Our results do not mean that reoperation is useless or harmful to patients with early progression. The worse PPS was primarily due to the more progressive characteristics of the tumor rather than reoperation. Therefore, cytotoxic agents in combination with reoperation may be more effective in improving clinical outcomes than reoperation alone. In fact, combining carmustine wafer implantation with surgical resection prolonged OS in newly diagnosed glioblastoma (47, 48). However, its effectiveness remains to be elucidated in recurrent GBM.

## Limitations

There are several limitations to the present study. First, heterogeneity existed in the pooled OS and PPS analyses. Heterogeneity in the OS analysis resulted from the study incorporating reoperation as a time-dependent covariate. Variation in adjusted confounding factors was the major cause for heterogeneity in PPS analysis. However, sensitive analysis supported the robustness of our primary findings. Second, only a few studies included reoperation as a time-dependent factor. Although all the included studies were of high quality, most of them are retrospective studies and evidence of prospective, randomized control trials was lacking in this meta-analysis. Third, we did not get the entirety of patient data in the three included studies; thus, univariable but not multivariable analyses were conducted. Both the landmark and Simon-Makuch methods have their own limitations, and the results cannot be used to make inferences (36, 49). For instance, the landmark method ignores the patient's status after the specific time and all deaths before that time. Fourth, the molecular marker profile, such as *MGMT* promoter methylation and *IDH1* mutation, is associated with clinical outcomes of GBM patients. Unfortunately, few studies incorporated these molecular markers into the multivariate Cox model (18, 22, 24). Fifth, the first-time treatment regimen and its feasibility may affect the decision of the treatment regimen at progression, which could lead to selection bias. In the case of patients with early recurrence, the chance to have radiotherapy and chemotherapy is low due to the short interval from the previous treatment. In addition, other treatment at recurrence may also influence the survival effects of reoperation. These concerns are not investigated in this study.

## Conclusions

The timing of reoperation may have an impact on the survival outcome in recurrent GBM patients, and survival benefits of reoperation in recurrent GBM may be overestimated when analyzed as fixed covariates. Proper analysis methodology should be used in future work to confirm the clinical benefit of reoperation.

## Author Contributions

Y-HZ collected data, performed the statistical analysis, and drafted the report. Z-YP participated in data collection analysis. DP, JD-F, and JP participated in the interpretation of the data and critically reviewed the manuscript. Z-FW and Z-QL designed the study and contributed to interpretation and discussion of the results. All authors read and approved the final manuscript.

### Conflict of Interest Statement

The authors declare that the research was conducted in the absence of any commercial or financial relationships that could be construed as a potential conflict of interest.

## References

[1] Alexander BM Cloughesy TF. Adult glioblastoma. J Clin Oncol. (2017) 35:2402–9. 10.1200/jco.2017.73.0119 28640706

[2] Stupp R Hegi ME Mason WP van den Bent MJ Taphoorn MJ Janzer RC. Effects of radiotherapy with concomitant and adjuvant temozolomide versus radiotherapy alone on survival in glioblastoma in a randomised phase iii study: 5-year analysis of the eortc-ncic trial. Lancet Oncol. (2009) 10:459–66. 10.1016/s1470-2045(09)70025-7 19269895

[3] Stupp R Mason WP van den Bent MJ Weller M Fisher B Taphoorn MJ. Radiotherapy plus concomitant and adjuvant temozolomide for glioblastoma. N Engl J Med. (2005) 352:987–96. 10.1056/NEJMoa043330 15758009

[4] Brandes AA Bartolotti M Franceschi E. Second surgery for recurrent glioblastoma: advantages and pitfalls. Expert Rev Anticancer Ther. (2013) 13:583–7. 10.1586/era.13.32 23617349

[5] Tosoni A Franceschi E Poggi R Brandes AA. Relapsed glioblastoma: treatment strategies for initial and subsequent recurrences. Curr Treat Options Oncol. (2016) 17:49. 10.1007/s11864-016-0422-4 27461038

[6] Weller M Cloughesy T Perry JR Wick W. Standards of care for treatment of recurrent glioblastoma–are we there yet? Neuro Oncol. (2013) 15:4–27. 10.1093/neuonc/nos273 23136223 PMC3534423

[7] Sanai N Berger MS. Surgical oncology for gliomas: the state of the art. Nat Rev Clin Oncol. (2018) 15:112–25. 10.1038/nrclinonc.2017.171 29158591

[8] Singapore Cancer Network (SCAN) Neuro-Oncology Workgroup Singapore cancer network (scan) guidelines for systemic therapy of high-grade glioma. Ann Acad Med Singapore. (2015) 44:463–73. 26763064

[9] Network NCC Nccn Clinical Practice Guidelines in Oncology, Version 1. (2018) Available online at: www.nccn.org/professionals/physician_gls/pdf/cns.pdf

[10] Boiardi A Silvani A Eoli M Lamperti E Salmaggi A Gaviani P. Treatment of recurrent glioblastoma: can local delivery of mitoxantrone improve survival? J Neurooncol. (2008) 88:105–13. 10.1007/s11060-008-9540-6 18283418

[11] Chen YR Sole J Ugiliweneza B Johnson E Burton E Woo SY. National trends for reoperation in older patients with glioblastoma. World Neurosurg. (2018) 113:e179–89. 10.1016/j.wneu.2018.01.211 29427817

[12] Delgado-Fernandez J Garcia-Pallero MA Blasco G Penanes JR Gil-Simoes R Pulido P. Usefulness of reintervention in recurrent glioblastoma: an indispensable weapon for increasing survival. World Neurosurg. (2017) 108:610–7. 10.1016/j.wneu.2017.09.062 28939537

[13] Skeie BS Enger PO Brogger J Ganz JC Thorsen F Heggdal JI. Gamma knife surgery versus reoperation for recurrent glioblastoma multiforme. World Neurosurg. (2012) 78:658–69. 10.1016/j.wneu.2012.03.024 22484078

[14] Wann A Tully PA Barnes EH Lwin Z Jeffree R Drummond KJ. Outcomes after second surgery for recurrent glioblastoma: a retrospective case-control study. J Neurooncol. (2018) 137:409–15. 10.1007/s11060-017-2731-2 29294233

[15] Zanello M Roux A Ursu R Peeters S Bauchet L Noel G. Recurrent glioblastomas in the elderly after maximal first-line treatment: does preserved overall condition warrant a maximal second-line treatment? J Neurooncol. (2017) 135:285–97. 10.1007/s11060-017-2573-y 28726173

[16] Chaichana KL Zadnik P Weingart JD Olivi A Gallia GL Blakeley J. Multiple resections for patients with glioblastoma: Prolonging survival. J Neurosurg. (2013) 118:812–20. 10.3171/2012.9.Jns1277 23082884 PMC3700339

[17] Stark AM Hedderich J Held-Feindt J Mehdorn HM Glioblastoma–the consequences of advanced patient age on treatment and survival. Neurosurg Rev. (2007) 30:56–61; discussion−2. 10.1007/s10143-006-0051-7 17119901

[18] Azoulay M Santos F Shenouda G Petrecca K Oweida A Guiot MC. Benefit of re-operation and salvage therapies for recurrent glioblastoma multiforme: results from a single institution. J Neurooncol. (2017) 132:419–26. 10.1007/s11060-017-2383-2 28374095

[19] Clarke JL Ennis MM Yung WK Chang SM Wen PY Cloughesy TF Is surgery at progression a prognostic marker for improved 6-month progression-free survival or overall survival for patients with recurrent glioblastoma? Neuro Oncol. (2011) 13:1118–24. 10.1093/neuonc/nor110 21813511 PMC3177665

[20] Filippini G Falcone C Boiardi A Broggi G Bruzzone MG Caldiroli D. Prognostic factors for survival in 676 consecutive patients with newly diagnosed primary glioblastoma. Neuro Oncol. (2008) 10:79–87. 10.1215/15228517-2007-038 17993634 PMC2600841

[21] Goldman DA Hovinga K Reiner AS Esquenazi Y Tabar V Panageas KS. The relationship between repeat resection and overall survival in patients with glioblastoma: a time-dependent analysis. J Neurosurg. (2018) 129:1231–39. 10.3171/2017.6.Jns17393 29303449 PMC6392195

[22] Nava F Tramacere I Fittipaldo A Bruzzone MG Dimeco F Fariselli L. Survival effect of first- and second-line treatments for patients with primary glioblastoma: a cohort study from a prospective registry, 1997-2010. Neuro Oncol. (2014) 16:719–27. 10.1093/neuonc/not316 24463354 PMC3984555

[23] Ortega A Sarmiento JM Ly D Nuno M Mukherjee D Black KL. Multiple resections and survival of recurrent glioblastoma patients in the temozolomide era. J Clin Neurosci. (2016) 24:105–11. 10.1016/j.jocn.2015.05.047 26671314

[24] Suchorska B Weller M Tabatabai G Senft C Hau P Sabel MC. Complete resection of contrast-enhancing tumor volume is associated with improved survival in recurrent glioblastoma-results from the director trial. Neuro Oncol. (2016) 18:549–56. 10.1093/neuonc/nov326 26823503 PMC4799687

[25] Woernle CM Peus D Hofer S Rushing EJ Held U Bozinov O. Efficacy of surgery and further treatment of progressive glioblastoma. World Neurosurg. (2015) 84:301–7. 10.1016/j.wneu.2015.03.018 25797075

[26] Tugcu B Postalci LS Gunaldi O Tanriverdi O Akdemir H. Efficacy of clinical prognostic factors on survival in patients with glioblastoma. Turk Neurosurg. (2010) 20:117–25. 10.5137/1019-5149.Jtn.2461-09.4 20401838

[27] Sastry RA Shankar GM Gerstner ER Curry WT. The impact of surgery on survival after progression of glioblastoma: a retrospective cohort analysis of a contemporary patient population. J Clin Neurosci. (2018) 53:41–7. 10.1016/j.jocn.2018.04.004 29680441

[28] McGirt MJ Chaichana KL Gathinji M Attenello FJ Than K Olivi A. Independent association of extent of resection with survival in patients with malignant brain astrocytoma. J Neurosurg. (2009) 110:156–62. 10.3171/2008.4.17536 18847342

[29] Park CK Kim JH Nam DH Kim CY Chung SB Kim YH. A practical scoring system to determine whether to proceed with surgical resection in recurrent glioblastoma. Neuro Oncol. (2013) 15:1096–101. 10.1093/neuonc/not069 23800677 PMC3714158

[30] Yong RL Wu T Mihatov N Shen MJ Brown MA Zaghloul KA. Residual tumor volume and patient survival following reoperation for recurrent glioblastoma. J Neurosurg. (2014) 121:802–9. 10.3171/2014.6.Jns132038 25061868

[31] Beyersmann J Gastmeier P Wolkewitz M Schumacher M. An easy mathematical proof showed that time-dependent bias inevitably leads to biased effect estimation. J Clin Epidemiol. (2008) 61:1216–21. 10.1016/j.jclinepi.2008.02.008 18619803

[32] Anderson JR Cain KC Gelber RD. Analysis of survival by tumor response. J Clin Oncol. (1983) 1:710–9. 10.1200/jco.1983.1.11.710 6668489

[33] Lu VM Jue TR McDonald KL Rovin RA. The survival effect of repeat surgery at glioblastoma recurrence and its trend: a systematic review and meta-analysis. World Neurosurg. (2018) 115:453–9.e3. 10.1016/j.wneu.2018.04.016 29654958

[34] Shamseer L Moher D Clarke M Ghersi D Liberati A Petticrew M. Preferred reporting items for systematic review and meta-analysis protocols (prisma-p) 2015: elaboration and explanation. BMJ. (2015) 350:g7647. 10.1136/bmj.g7647 25555855

[35] Cota GF de Sousa MR Fereguetti TO Rabello A. Efficacy of anti-leishmania therapy in visceral leishmaniasis among hiv infected patients: a systematic review with indirect comparison. PLoS Negl Trop Dis. (2013) 7:e2195. 10.1371/journal.pntd.0002195 23658850 PMC3642227

[36] Dafni U. Landmark analysis at the 25-year landmark point. Circ Cardiovasc Qual Outcomes. (2011) 4:363–71. 10.1161/circoutcomes.110.957951 21586725

[37] Windschitl H Scott M Schutt A McCormack G Everson L Cullinan S. Randomized phase ii studies in advanced colorectal carcinoma: a north central cancer treatment group study. Cancer Treat Rep. (1983) 67:1001–8. 6640551

[38] Simon R Makuch RW. A non-parametric graphical representation of the relationship between survival and the occurrence of an event: application to responder versus non-responder bias. Stat Med. (1984) 3:35–44. 6729287 10.1002/sim.4780030106

[39] Kim HR Kim KH Kong DS Seol HJ Nam DH Lim DH. Outcome of salvage treatment for recurrent glioblastoma. J Clin Neurosci. (2015) 22:468–73. 10.1016/j.jocn.2014.09.018 25595963

[40] Tully PA Gogos AJ Love C Liew D Drummond KJ Morokoff AP. Reoperation for recurrent glioblastoma and its association with survival benefit. Neurosurgery. (2016) 79:678–89. 10.1227/neu.0000000000001338 27409404

[41] Goldman DA Panageas KS. Letter to the editor: biases in estimation of overall survival in patients who underwent repeat resection of glioblastoma. J Neurosurg. (2016) 125:519–22. 10.3171/2015.11.Jns152515 27257842

[42] Fisher LD Lin DY. Time-dependent covariates in the cox proportional-hazards regression model. Annu Rev Public Health. (1999) 20:145–57. 10.1146/annurev.publhealth.20.1.145 10352854

[43] Bolard P Quantin C Esteve J Faivre J Abrahamowicz M. Modelling time-dependent hazard ratios in relative survival: Application to colon cancer. J Clin Epidemiol. (2001) 54:986–96. 11576809 10.1016/s0895-4356(01)00363-8

[44] Jatoi I Anderson WF Jeong JH Redmond CK. Breast cancer adjuvant therapy: time to consider its time-dependent effects. J Clin Oncol. (2011) 29:2301–4. 10.1200/jco.2010.32.3550 21555693 PMC3107746

[45] Malcovati L Germing U Kuendgen A Della Porta MG Pascutto C Invernizzi R. Time-dependent prognostic scoring system for predicting survival and leukemic evolution in myelodysplastic syndromes. J Clin Oncol. (2007) 25:3503–10. 10.1200/jco.2006.08.5696 17687155

[46] Cecchi E Ciccone G Chirillo F Imazio M Cecconi M Del Ponte S. Mortality and timing of surgery in the left-sided infective endocarditis: an italian multicentre study. Interact Cardiovasc Thorac Surg. (2018) 26:602–9. 10.1093/icvts/ivx394 29272391

[47] Westphal M Hilt DC Bortey E Delavault P Olivares R Warnke PC. A phase 3 trial of local chemotherapy with biodegradable carmustine (bcnu) wafers (gliadel wafers) in patients with primary malignant glioma. Neuro Oncol. (2003) 5:79–88. 10.1093/neuonc/5.2.79 12672279 PMC1920672

[48] Roux A Peeters S Zanello M Bou Nassif R Abi Lahoud G Dezamis E. Extent of resection and carmustine wafer implantation safely improve survival in patients with a newly diagnosed glioblastoma: a single center experience of the current practice. J Neurooncol. (2017) 135:83–92. 10.1007/s11060-017-2551-4 28669011

[49] Schultz LR Peterson EL Breslau N. Graphing survival curve estimates for time-dependent covariates. Int J Methods Psychiatr Res. (2002) 11:68–74. 10.1002/mpr.124 12459796 PMC6878542

