# Supplementary material for: A Meta-Analysis of Survival Outcomes Following Reoperation in Recurrent Glioblastoma: Time to Consider the Timing of Reoperation
Source: Front Neurol. 2019 Mar 26;10:286. doi: 10.3389/fneur.2019.00286 (PMC6448034; doi:10.3389/fneur.2019.00286)

Supplemental information

Supplemental Table S1 Quality assessment of included studies using the Newcastle–Ottawa Scale for cohort studies

Study	Selections				Comparability	Outcome			Score
	A	B	C	D	E	F	G	H	
Azoulay et al., 2017	1	1	1		1	1	1	1	7
Boiardi et al., 2008	1	1			2	1	1		6
Chaichana et al., 2013	1	1	1		1	1	1	1	7
Chen et al., 2018	1	1	1		1	1	1		6
Clarke et al., 2011	1	1	1		1	1	1		6
Delgado-Fernandez et al., 2017	1	1	1		1	1	1	1	7
Filippini et al., 2008 (I)	1	1	1		2	1	1	1	8
Filippini et al., 2008 (II)	1	1	1		2	1	1	1	8
Goldman et al., 2018	1	1	1		2	1	1	1	8
Nava et al., 2014 (I)	1	1	1	1	2	1	1	1	9
Nava et al., 2014 (II)	1	1	1	1	2	1	1	1	9
Ortega et al., 2016	1	1	1		1	1	1		6
Skeie et al., 2012	1	1	1		2	1	1	1	8
Suchorska et al., 2016	1	1	1	1	1	1	1	1	8
Tully et al., 2016	1	1	1		1	1	1	1	7
Tugcu et al., 2010	1	1	1		1	1	1	1	7
Woernle et al., 2015	1	1	1		1	1	1		6
Zanello et al., 2017	1	1	1		1	1	1	1	7
Kim et al., 2015	1	1	1		2	1	1	1	8
Stark et al., 2007	1	1	1		1	1	1		6
McGirt et al., 2009	1	1	1		2	1	1		7
Sastry et al., 2018	1	1	1		2	1	1	1	8
Wann et al., 2018	1	1	1		1	1	1		6

A, representativeness of the exposed cohort: highly representative or somewhat representative (one star); no description (no score). B, selection of the non-exposed cohort: patients drawn from the same community as the exposed cohort (one score); patients drawn from a different source or no description (no score). C, ascertainment of exposure: information collected from secure record or structured interview (one score); no description (no score). D, demonstration that outcome of interest was not present at start of study: yes (one score), no (no score). E, comparability of cohorts on the basis of the design or analysis: study controls for important factor, time to recurrence, *MGMT* promoter status/*IDH1* mutation, initial extend of resection, age, treatment (two scores: time to recurrence is essential, at least other 3 factor was included, one star: time to recurrence was ignored, and some of them were included.). F, assessment of out-come: independent blind assessment or record linkage (one score); self-report or no description (no star). G, follow-up long enough for outcomes to occur: yes (one score); no (no score). H, adequacy of follow-up of cohorts: complete follow-up (one score); follow-up rate\80% and no description of those lost (no score); no statement (no score).

Supplemental Figure S1 Egger’s test to evaluate the publication bias for overall survival (A) and post-progression survival (B).

A

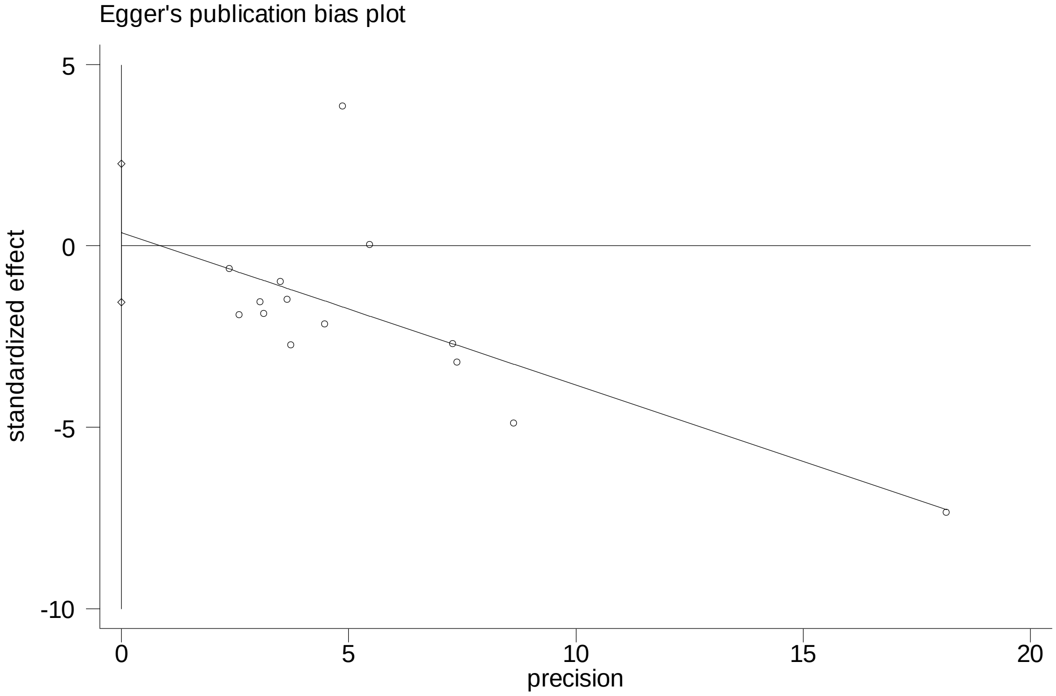

B

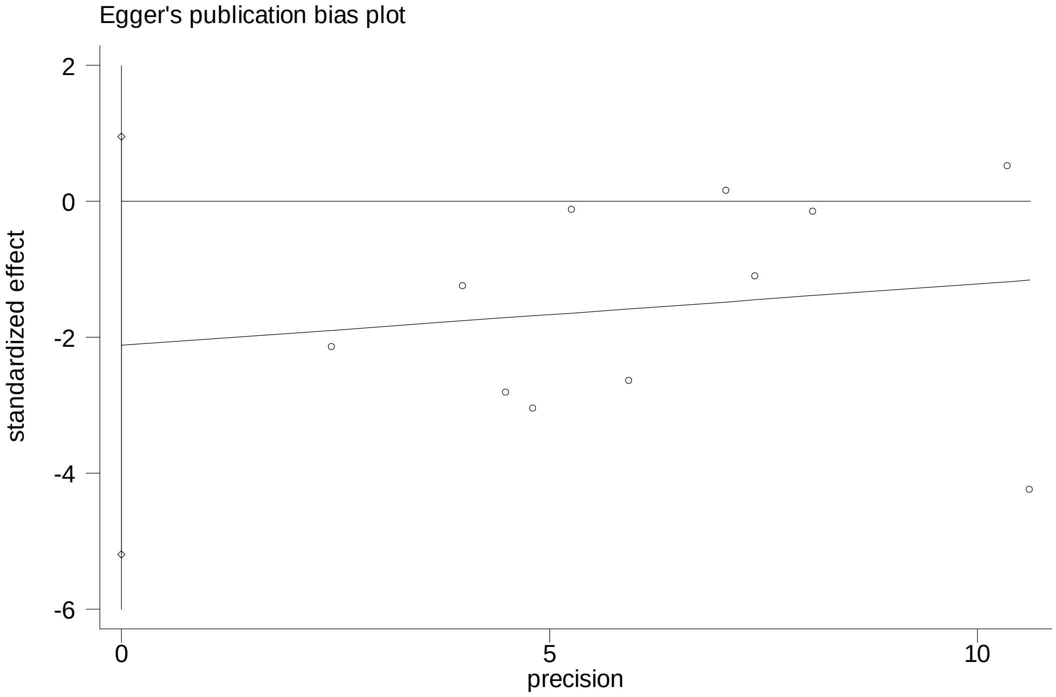

Supplemental Figure S2 Sensitivity analysis for overall survival (A) and post-progression survival (B).

A

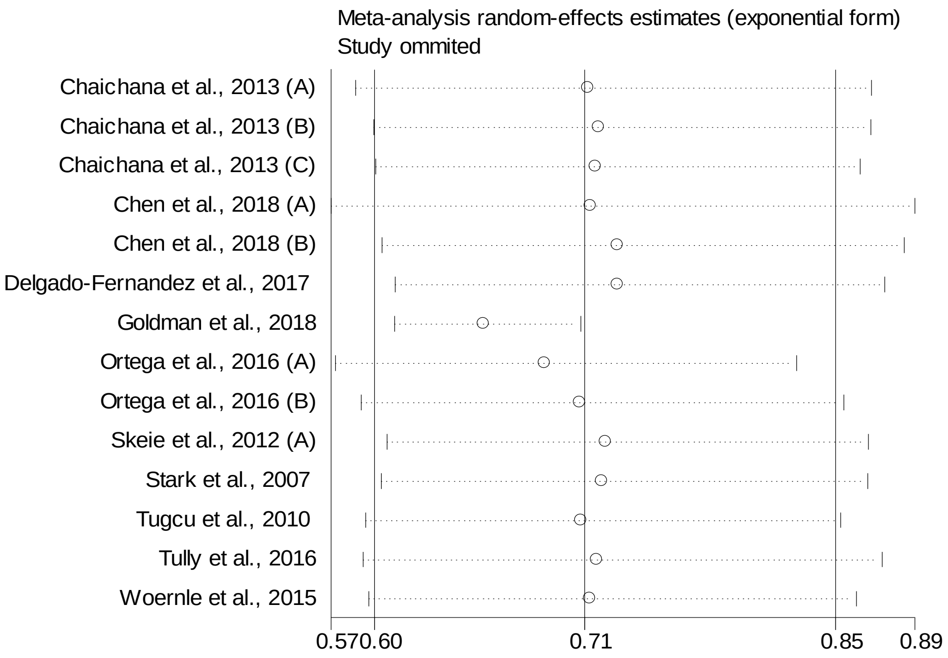

B

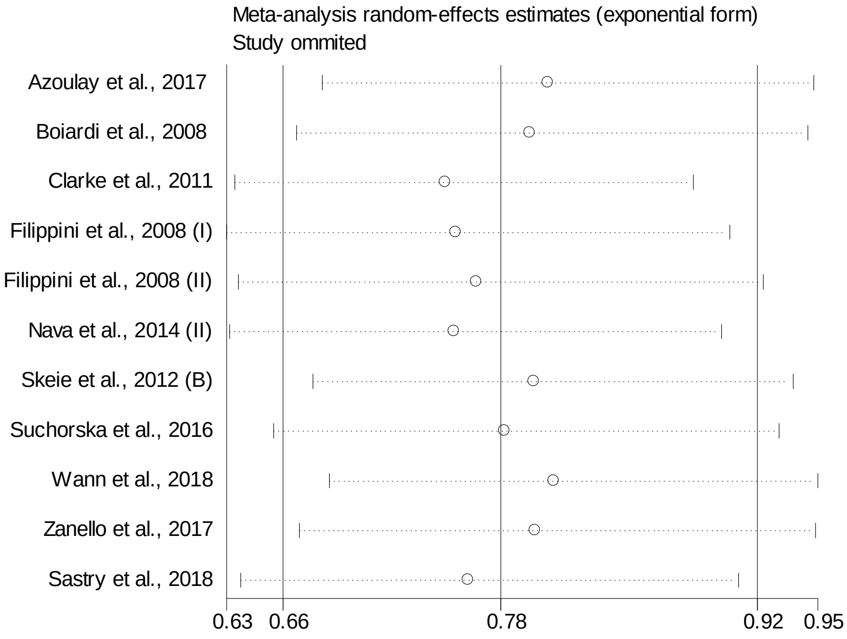

Supplemental Figure S3 Forest plots for hazard ratios comparing OS in patients who underwent one reoperation vs multiple reoperation.

Study ID	HR for OS (95% CI)	Favors resection / Favors non-resection	Weight%
One Reoperation			
Chaichana et al., 2013 (A)	0.69(0.53-0.90)		9.81
Chen et al., 2018 (A)	0.67(0.60-0.74)		12.10
Delgado-Fernandez et al.,	0.48(0.28-0.81)		6.03
Goldman et al., 2018	2.19(1.47-3.27)		7.67
Ortega et al., 2016 (A)	1.00(0.70-1.43)		8.36
Skeie et al., 2012 (A)	0.48(0.22-1.01)		3.85
Stark et al., 2007	0.55(0.30-1.02)		4.95
Tugcu et al., 2010	0.76(0.34-1.72)		3.42
Tully et al., 2016	0.65(0.50-0.84)		9.87
Woernle et al., 2015	0.66(0.39-1.13)		5.87
Subtotal(I^2=77.3%, p<0.001)	0.75(0.59-0.95)		71.9
Multiple Reoperation			
Chaichana et al., 2013 (B)	0.61(0.40-0.95)		7.16
Chaichana et al., 2013 (C)	0.60(0.32-1.14)		4.79
Chen et al., 2018 (B)	0.57(0.45-0.71)		10.4
Ortega et al., 2016 (B)	0.75(0.43-1.31)		5.62
Subtotal(I^2=0%, p=0.835)	0.59(0.50-0.71)		28.0
Overall(I^2=70.0%, p<0.001)	0.71(0.60-0.85)		100.

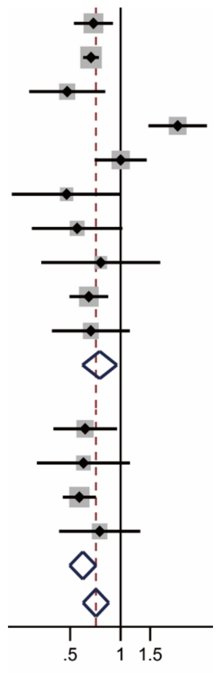

Supplement: Supplementary file 1 [file Data_Sheet_1.PDF]
